# Social cognition, psychosocial development and well-being in galactosemia

**DOI:** 10.1186/s13023-024-03335-2

**Published:** 2024-09-06

**Authors:** Clémentine Bry, Klervi Propice, Jessica Bourgin, Morgane Métral

**Affiliations:** Univ. Savoie Mont Blanc, Univ. Grenoble Alpes, LIP/PC2S, Grenoble, 38000 France

**Keywords:** Galactosemia, Social cognition, Adults, Theory of mind, Emotion recognition

## Abstract

**Background:**

Classic galactosemia is a rare inherited metabolic disease with long-term complications, particularly in the psychosocial domain. Patients report a lower quality of social life, difficulties in interactions and social relationships, and a lower mental health. We hypothesised that social cognition deficits could partially explain this psychological symptomatology. Eleven adults with galactosemia and 31 control adults participated in the study. We measured social cognition skills in cognitive and affective theory of Mind, and in basic and complex emotion recognition. We explored psychosocial development and mental well-being.

**Results:**

We found significant deficits on all 4 social cognition measures. Compared to controls, participants with galactosemia were impaired in the 2nd-order cognitive theory of mind, in affective theory of mind, and in basic and complex emotion recognition. Participants with galactosemia had a significant delay in their psychosexual development, but we found no delay in social development and no significant decrease in mental health.

**Conclusion:**

Social cognition processes seem impaired among our participants with galactosemia. We discuss the future path research may follow. More research is needed to replicate and strengthen these results and establish the links between psychosocial complications and deficits in social cognition.

**Supplementary Information:**

The online version contains supplementary material available at 10.1186/s13023-024-03335-2.

## Introduction

Classic galactosemia (OMIM #230400) is a rare genetic disorder with a prevalence of 1:19000 to 1:44000 [1], due to a deficiency of Galactose-1-phosphate Uridyltransferase in the Leloir Pathway (GALT; EC2.7.712). The galactose-free diet is lifesaving and resolves the acute neonatal clinical picture. However, despite the galactose-free diet, long-term complications are frequent and vary massively between individuals [2]. They appear at the levels of fertility, neurological, and bone health but also at psychological levels [3–7]. The complications are multifactorial (e.g., metabolite toxicity, aberrant glycosylation, myo-inositol deficiency, ER stress, oxidative stress, structural impairment), and some mechanisms are yet to be explored (e.g., epigenetic effects and genetic modifiers) [8].

### Psychosocial profile

Galactosemia negatively impacts the quality of life of the patients on cognitive, social and communication functions. People with galactosemia attend special schools more often and reach a lower level of education and employment than their siblings [4, 5, 9]. They suffer from delays in their social and psychosexual developments [9, 10]. More than 90% of the patients reported social impacts of galactosemia, with difficulties in social interactions and relationships and feelings of social isolation [11]. Fishler et al. [12, 13] and Lee [14] reported emotional disturbances with excessively shy and anxious children. Relatedly, patients are more likely to stay single, living with their parents or under supervision, and to not have children than healthy people or with other metabolic conditions [4, 9, 10, 15].

Mental health and behavioural issues are also frequently reported, notably anxiety and depression disorders [2, 7].

### Neurocognitive profile

Psychosocial complications could be related to neurocognitive deficits, since many dysfunctional processes are typically found in galactosemia. Galactosemia is associated with a global impairment of cognitive functioning. The global intelligence quotient is generally below average (mean IQ = 87, range [47–122]) [16]. A large number of neurocognitive processes are somewhat impaired in patients, again with a wide diversity between individuals (i.e., language, verbal and visual memory, attention, processing speed, inhibition and flexibility). However, other neurocognitive processes - social cognition processes that have not yet been thoroughly explored in galactosemia - are also good candidates to explain the psychosocial complications. Indeed, Beauchamp and Anderson [17] proposed that social cognition contributes to social functioning and must be considered separately from cold neurocognitive processes. We contend that specific deficits in social cognition processes could be at stake in psychosocial complications.

### Social cognition

Social cognition is the ability to recognise, process and use socially relevant information to adapt social behaviour [18]. It encompasses several processes, including recognition of emotional facial expressions and theory of mind [19]. The recognition of emotional facial expressions is the ability to perceive and correctly identify the emotions displayed by others. The Theory of Mind (ToM) is the ability to make inferences about the mental states (beliefs, intentions, and emotions) of others [20]. These two processes are central to social interactions, as they allow understanding subtle social signals [21] and regulating social interactions effectively. Deficits in these processes are known to occur in certain pathologies such as autism spectrum disorder [22, 23].

Korner et al. [24] showed for the first time that 22 Swiss patients with galactosemia had a significant deficit in an Emotion Recognition Task (ERT) and in rapid visual information processing (RVP). The authors note that ERT and RVP deficits may be related to subclinical autism spectrum disorders (ASDs) in galactosemia. More recently, Hermans et al. [25] also investigated social cognition in a Dutch galactosemia sample of 12 children (8–17 years old) and 11 adults (18–52 years old) without cognitive deficits (IQ > 70). They measured cognitive theory of mind, cognitive and affective empathy and basic emotion recognition. They found impairments on emotion recognition of disgust, fear, happiness, and sadness, but no impairment on anger and surprise. They also found deficits in the cognitive theory of mind. Adults showed a significant decrease in cognitive and affective empathy and children in affective empathy.

Consistent with this trend of social cognition investigation in galactosemia, we want to deepen the understanding of social cognition deficits and their links with psychosocial development and mental well-being.

Theory of mind is a metacognitive ability to understand the thoughts (beliefs, intentions, cognitions) and the affective states (emotions, feelings) of others. Affective and cognitive theories of mind are somewhat independent components: they recruit different neurological circuits, and they can be differently impaired (e.g., in ASD, in schizophrenia, in Alzheimer’s disease). They are high-order processes that require both information decoding (perceptual processing) and reasoning. Hence, they can be impaired even when intellectual resources are preserved, and they can be preserved even when intellectual resources are impaired, as in Down syndrome. Finally, theories of mind can also be distinguished between first-order and second-order representations. First-order representations require ‘mere’ perspective taking when second-order representations require the adoption of two different perspectives at the same time. Second-order representations recruit more executive resources than first-order representations (See [26] for a review).

There are numerous tests of theory of mind, some measuring only one type of mental states, only epistemic mental states like in false beliefs tasks or only affective states like in emotion recognition tasks, and others measuring mixed mental states like in the faux-pas task where one has to understand both what is wrong in the situation (cognitive ToM) and how it can hurt the feelings of a character (affective ToM). In this study, we will use both a false-belief task, distinguishing between first and second-order mentalisations and a mixed task requiring both cognitive and affective mentalisation.

Emotion recognition can be considered a lower-order process requiring mostly perceptual processing and less reasoning than theory of mind tasks. A frequent distinction made in emotion recognition tasks refers to basic vs. complex emotions. The basic emotions (joy, surprise, anger, disgust, sadness and anger) are thought to be innate and universal when complex emotions are thought to express a cognitive state (e.g., thoughtful, tired, preoccupied…) or a social state (e.g., charming, guilty, friendly). These complex emotions would not be predetermined but socially acquired. Recognition of basic emotion expressions have been tested in galactosemia samples but not the recognition of complex emotions. In this study we will use both types of tests in this online study.

### The present study

Patients with galactosemia are on average impaired at the cognitive level, with very inconsistent deficits across patients. On a psychological level, they suffer from isolation, fewer social and intimate bonds. They are described as shy and socially unadjusted. They are also at risk for mental disorders, such as anxiety and depression. In this online study run between March and August 2022, we tested whether social cognition deficits could partially explain this psychological vulnerability.

## Method

### Participants

The control group comprised 26 women and 5 men aged between 20 and 59 years old (M = 27.8, SD = 10.2). The Galactosemia group consisted of 8 women and 3 men between 18 and 47 years old (M = 30.4, SD = 9.62). The inclusion criteria for the galactosemia group were having classic galactosemia, being 18 years old or older and French-speaking. We did not have the means to control intellectual and executive functions (the study would have been too long and too taxing for the participants). However, the social cognition tasks include control questions that partial out possible intellectual deficits in the measured performance. We did not ask participants about their residual enzymatic activity level, metabolite levels and genotype. We did not have specific hypotheses related to these sensitive health data, so we complied with the French regulation of non-medical research and the recommendations for good-research practices in psychology and did not collect these data. However, we can assume with good confidence that all participants with galactosemia were diagnosed through clinical symptoms and have classic galactosemia since galactosemia is not yet included in newborn screening in France and the patient organisation has only members with classic galactosemia.

## Material

### Social cognition: theory of mind

#### Cognitive theory of mind

We used the TOM-15 task [27] to assess the ability to deduce others’ mental states (false belief task). This task was validated among 175 healthy French adults. It consists of 15 stories of 3-vignette strips, each related to a two-choice question. Eight stories present first-order false beliefs and seven stories present second-order false beliefs. The first order ToM refers to the representations one can have about the mental state of another person (i.e., *I think that you think*…). The 2nd order ToM is more complex and refers to the representations one can have about the mental state another person has about the representations of a third person (i.e., *I think that you think that s/he thinks*…). At the end of the study, the 15 stories are presented again, with a control understanding question. Consistent with the scoring recommendations, the correct ToM answers received 1 point (maximum = 15) and we considered the ToM answers only for perfectly understood stories (when the participant gave the correct answer to the control understanding question). We selected this task because the stimuli are easily displayed on computers and attractive (colourful comic strips). The control questions allow controlling the impact of possible intellectual deficits within the theory of mind performance. Finally, participants select one out of two choice answers; they do not have to give verbal answers which may be a hurdle for people with language issues.

#### Affective theory of mind

We use the French short version of the faux-pas test [19, 28] to assess the mentalisation of affective states. This task is part of the Bordeaux protocol for evaluating social cognition (PECS-B, 19) and the MiniSEA [29]. It consists of five stories in which a character commits a ‘faux-pas’ (the character unintentionally offends another character) and five stories in which there are no ‘faux-pas’. The stories were presented in written form and were also played once in an audio format. The participants answer several questions regarding whether there is a faux-pas or not and who commits the faux-pas (detection), why it is a faux-pas (inappropriateness), whether the faux-pas was intentional or not (intention), what the character believes (belief) and what emotions the target character feels (empathy). Two control questions about the story understanding are also used. Consistent with the scoring recommendations, correct answers received 1 point (maximum = 30 points on the Faux-Pas stories) and we considered the Faux-Pas answers only to perfectly understood stories (when the participant gave the correct answers to the two understanding questions). Correct answers to control stories (when no faux-pas) receive 2 points (Max = 10 points) and again we considered the answers only when the stories were perfectly understood. This scoring allows assessing the theory of mind performance controlling for general intellectual deficit.

### Social cognition: recognition of emotional facial expression

#### Basic and dynamic emotional facial expressions

We use the dynamic version of Karolinska Directed Emotional Faces database [30, 31]. It consists of 1 033 milliseconds videos of 40 faces going from neutral to an emotional expression (i.e., joy, anger, sadness, surprise, disgust and fear). In our study, participants were presented with two female and two male stimuli for each emotion, randomly selected from the database. Each video was looped to the first neutral face image and could be played only once to mimic natural visual information processing (i.e., they had only one second to visually process the emotional information). Participants chose which emotion was presented among the 6 basic emotions. Correct identification was awarded 1 point (max = 24).

#### Complex and static emotional facial expressions

We used the French adult version of the Reading the Mind in the Eye Test (RMET), which consists of 36 pictures of the eye area from emotional faces [32, 33]. Each black and white picture is presented with four emotional labels, and participants choose the most relevant to describe the person’s state of mind. This task is part of the Bordeaux protocol for evaluating social cognition [19]. The correct identification was awarded 1 point (max = 36). In the general population controls, the average recognition was M = 26.2, SD = 3.6. In a student sample, the average recognition was M = 28, SD = 3.5 [27]. The data of our control group are similar (M = 27.03, SD = 3.48).

### Well-being and psychosocial development

#### Well-being

We used the French version of the Warwick-Edinburgh Mental Well-being Scale [34, 35] to measure psychological well-being. Fourteen items measure the frequency of positive feelings and functioning from Never [1] to All the time [5]. Scores can range from 14 to 70. The mean score in a nonclinical French sample of workers was 51.47 (SD = 7.19) and the mean score in a non-clinical French sample of students was 51.88 (SD = 6.87) before the Covid-19 crisis. The data of our control group are similar (M = 50.4, SD = 8.73).

#### Psychosocial development

We translated and adapted the Course of Life Questionnaire to French [36]. It measures whether the individual reaches the milestones of social development, psychosexual development, autonomy, substance abuse and antisocial behaviour, throughout childhood, teenage, adulthood and whole life.

Each item of the Course of Life Questionnaire is usually coded Fail/Reach the milestone. However, we wanted to have more nuance with regard to the delay and awarded increasing points to the different option answers. For example, the question “how many friends did you usually have at school’ was coded 0 when the answer was ‘zero’, 1 for “1 friend”, 2 for “2 or 3 friends’ and 3 for “4 friends or more”. The higher the score, the better/earlier the milestone is reached.

The social development score varies between 0 and 23; the Autonomy Development score varies between 0 and 15; the psychosexual development scores between 0 and 12, the substance abuse scores between 0 and 34, the antisocial behaviour score between 0 and 5.

### Procedure

#### Recruitment and sign-up

Participants with galactosemia were recruited through the Facebook pages of the patients’ French association and by email to the members of the patients’ French association. Control participants were recruited among the siblings of the patients and the general population through non-sponsored Facebook publications on the experimenters’ personal pages. The recruitment messages stated the target characteristics (adults with or without GALT), the main aim (social cognition and well-being), the estimated completion time (about 30 to 60 min), the retribution for participation (10€ voucher) and how to connect to the online study platform. The homepage page of the platform provided all the needed information to form informed consent. Volunteers created an account on the platform, then read a formal information notice and agreed with the consent form before starting the study, set up on Limesurvey [37]. Participants were allowed to stop and resume later if needed. This allowed participants to manage their fatigue, the study being quite long (average completion time ~ 57 min, SD ~ 20 min) and the tasks quite demanding.

#### Order of the tasks

Participants first indicated their gender (F or M)^1^, whether they had galactosemia, another condition or none, and their year of birth. Then participants answered (1) the Warwick-Edinburgh Mental Wellbeing Scale (WEMWBS), (2) the first-order cognitive Theory of Mind task (ToM15), (3) the Childhood Course of Life questions, (4) the second-order cognitive Theory of Mind task (ToM15), (5) the adolescence Course of Life questions, (6) the complex emotion recognition task (RMET), (7) the Adulthood Course of Life questions, (8) the affective Theory of Mind (Faux-Pas test), (9) the whole-life Course of Life questions, (10) the basic emotions recognition test (KDEF-Dyn) and finally 11) the comprehension questions of the ToM15 test. The participants then read a short closing note (customary in psychology research) and received their voucher code.

We have some incomplete data for 4 control and 1 galactosemia participants as the study was quite long and these participants interrupted their participation before the end. We kept all participants in the file, and the headcount may differ between variables (we did not replace the missing values).

### Hypotheses

We hypothesised that participants with galactosemia would score lower than controls on all social cognition tasks. Also, we expected that they would reach less milestones on social development and psychosexual development. We expected participants with galactosemia would have a lower mental well-being than the control participants.

For participants with galactosemia, we expected the social cognition scores to correlate positively with the psychosocial and well-being scores.

## Results

### Statistical analyses

All tasks were coded following recommendations, by the authors - all trained psychologists. Because all measures and tasks have different codings, minimum and maximum, we transformed all raw scores into percentages to ease the interpretation of the results (100% = the maximum score one can get on the task). The higher the better the performance. Because of non-normality of distribution and heterogeneous variance, we used Mann-Whitney tests comparing participants with galactosemia to control participants in a unilateral test^2^. We also report Cohen’s d as an indication of the size effect. All descriptive statistics are reported in Table 1. Statistical analyses were run with Jamovi software [38], with the JMV package.

**Table 1:** Scores and statistics for social cognition tasks, well-being and psychosocial measures, for galactosemia and control participants

		Participants with galactosemia					Control participants							
		M	SD	range	Med	*N*	M	SD	range	Med	*N*	d	Stat	*p*
TOM15	**Understanding**	**92**	**8.2**	**80–100**	**93.3**	10	**97.3**	**3.34**	**93.3–100**	**100**	27	**0.84**	**87**	**0.035**
	1st-order ToM	79.9	31.6	14.3–100	100	10	93.8	10.4	62.5–100	100	27	0.59	112.5	0.18
	**2nd-order ToM**	**66**	**35.4**	**0–100**	**71.4**	**10**	**90.5**	**16.5**	**28.6–100**	**100**	**27**	**0.88**	**75**	**0.013**
Faux-Pas	**Understanding**	**93**	**11.1**	**70–100**	**97.5**	10	**98.9**	**2.12**	**95–100**	**100**	27	**0.74**	**91.5**	**0.033**
	**Test stories**	**75.6**	**17.1**	**33.3–93.3**	**78.3**	**10**	**83.2**	**15.3**	**33.3–96.7**	**100**	**27**	**0.47**	**85.5**	**0.046**
	Control stories	95	15.8	20–100	100	10	94.1	17.4	50–100	100	27	− 0.06	129.5	0.63
KDEF-Dyn	**recognition**	**64.2**	**13.3**	**37.5–83.3**	**64.4**	**10**	**78.7**	**7.15**	**58.3–87.5**	**79.2**	**27**	**1.36**	**42**	**0.001**
RMET	**recognition**	**58.3**	**19.1**	**22.2–86.1**	**61.1**	11	**75.1**	**9.66**	**52.8–88.9**	**75**	29	**1.11**	**63**	**0.002**
	**time (seconds)**	**548**	**354**	**68–1308**	**507**	11	**340**	**181**	**69–1104**	**311**	**29**	**− 0.74**	**78**	**0.013**
WEMWBS	Well-being	46	9.58	31–60	49	11	50.4	8.73	31–69	51	31	0.49	1.39	0.087
Course of Life questionnaire	**Psycho-sexual**	**43.3**	**27.2**	**0-83.3**	**50**	**10**	**59.6**	**13.2**	**33–75**	**66.7**	**27**	**0.76**	**81.5**	**0.032**
	Social	63.8	16.6	41–85.4	67.5	11	58.6	18.3	17–88	62	28	− 0.27	140	0.77
	Autonomy*	47.2	22.1	17–89	47.2	10	56.4	25.6	0–94.4	55.5	27	0.38	102	0.13
	Anti-social	23.5	12.3	17–50	16.7	11	23.3	15.5	17-83.3	16.7	30	− 0.01	156.5	0.64
	Substance abuse	15	14.3	2.9–47.1	8.82	11	21	15.8	0–64.7	20.6	28	0.40	145.5	0.14

* One control male participant of 30 years old has a null score in autonomy which can be considered as an outlier data. He was not an outlier on any other measure. When we remove this participant from the autonomy analysis, it does not change the results (d = 0.49, *p* = .092)

### Social cognition

#### Cognitive theory of mind (ToM15 task)

Participants with galactosemia scored significantly lower on the understanding questions than control participants, *d* = 0.84, *U* = 87, *p* = .035. Given the difference in understanding the scenarios and our will to control for potential cognitive deficits, we computed the ToM scores only with ToM answers from perfectly understood scenarios. The first-order ToM score of participants with galactosemia is not significantly lower to control participants’ score, *d* = 0.591, *U* = 112.5, *p* = .18. However, the second-order ToM score of participants with galactosemia is significantly lower to control participants’ score, *d* = 0.88, *U* = 75, *p* = .013.

#### Affective theory of mind (faux-pas task)

The understanding score of participants with galactosemia is lower than control participants’ score, *d* = 0.736, *U* = 91.5, *p* = .033.

Given the differences in understanding scenarios, consistently with the scoring recommendations and our will to control for potential cognitive deficits, the scores were computed only with the ToM answers from perfectly understood scenarios. Regarding the control stories, the two groups have similar performances (they understand there is no faux-pas), *d* = − 0.05, *U* = 129.5, *p* = .635. However, regarding the stories with a ‘faux-pas’, participants with galactosemia scored significantly lower than control participants on all combined dimensions, *d* = 0.467, *U* = 85.5, *p* = .046.

The Faux-Pas task is a mixed-task with different dimensions (cognitive, affective, volitional mental states). It is the empathy dimension that is significantly impaired among participants with galactosemia, *d* = 0.52, *U* = 79.5, *p* = .02 (See Table 2).

**Table 2:** Scores and statistics for each dimension on the affective theory of mind task and for each emotion on the Basic emotion recognition task, by pathology group

		Participants with galactosemia				Control participants				d	Stat	p
		N	M	SD	Med	N	M	SD	Med			
Faux-Pas	Detection	10	90.8	9.2	100	27	94.2	13.7	100	0.20	125	0.33
	Inappropriateness		76.0	24.9	77.5		85.6	19.9	100	0.42	101.5	0.11
	Intention		48.0	29.4	50		59.8	24.4	60	0.44	98.5	0.11
	Beliefs		74.0	20.7	80		79.6	17.4	80	0.29	118	0.27
	**Empathy**		74.0	19.4	77.5		85.7	25	100	**0.52**	**79.5**	**0.02**
Basic Emotion Recognition	Joy	10	3.4	0.84	4	27	3.78	0.51	4	0.54	103	0.075
	**Anger**		**2.6**	**0.84**	**3**		**3.41**	**0.57**	**3**	**1.12**	**62.5**	**0.003**
	**Fear**		**1.4**	**0.97**	**1.5**		**2.81**	**0.83**	**3**	**1.57**	**39**	**0.001**
	**Disgust**		**2.3**	**0.95**	**2**		**2.93**	**0.78**	**3**	**0.72**	**85**	**0.037**
	Surprise		3.3	1.06	4		3.26	0.86	4	− 0.04	126.5	0.63
	Sadness		2.4	1.35	2		2.7	0.91	3	0.26	117	0.27

#### Recognition of dynamic basic facial emotions (KDEF-Dyn)

Participants with galactosemia performed significantly less than control participants, *d* = 1.36, *U* = 42, *p* = .001.

Compared to control participants, fear appears to be the most impaired item (*d* = 1.57), followed by anger (*d* = 1.12) and disgust (*d* = 0.72). Joy (*d* = 0.54), sadness (*d* = 0.26), and surprise (*d* = − 0.04) seem to be mostly preserved with correct levels of identification (see Table 2).

#### Recognition of complex emotional facial expressions (RMET)

Participants with galactosemia significantly underperformed compared to control participants, *d* = 1.11, *U* = 63, *p* = .002. Participants with galactosemia spent more time on the task than control participants, *d* = − 0.74, *U* = 78, *p* = .013, bilateral test.

**Fig. 1:**
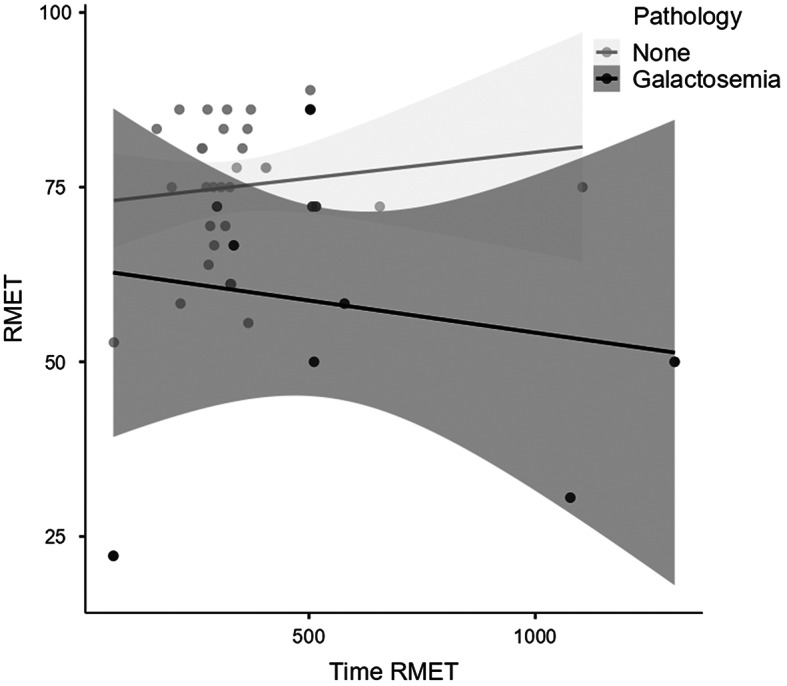
Scatterplot of RMET performance and time for galactosemia and control participants

The scatterplot of the performance of RMET by time spent on the task (Fig. 1) shows that the control participants had better performance when they spent more time on the task. However, participants with galactosemia did not increase their performance when they spent more time on the task.

### Well-being and development

#### Well-being

Well-being, measured by the WEMWBS, is distributed normally so we ran a parametric unilateral Student T test. The well-being of participants with galactosemia is only marginally lower than control participants, *d* = 0.486; *t* [40] = 1.39, *p* = .087.

#### The course of life questionnaire

The psychosexual development of participants with galactosemia is significantly delayed compared to the control participants, *d* = 0.76, *U* = 81.5, *p* = .032. We found no differences in social and autonomy development scores, antisocial, and substance abuse scores.

### Correlation between social cognition, well-being and development in galactosemia

In order to describe the potential links between social cognition and well-being and development in galactosemia, we computed a Spearman correlation matrix (Table 3), for the participants with galactosemia only.

**Table 3:** Spearman correlations between social cognition, well-being and psychosocial development measures for participants with galactosemia only

	Well-being	Psycho-sexual	Social	Autonomy	Substance abuse	Antisocial	ToM15 1st-order	ToM15 2nd-order	ToM15 understand	Affective ToM	Aff.ToM understand	Basic ERT	
Psycho-sexual	-0.133	—											
Social	0.442	0.480	—										
Autonomy	0.224	0.164	0.021	—									
Substance abuse	-0.359	0.431	-0.192	0.106	—								
Antisocial	-0.061	-0.543	-0.400	0.513	0.196	—							
ToM15 1st-order	-0.010	0.373	0.195	0.498	0.421	0.015	—						
ToM15 2nd-order	0.156	0.428	0.523	0.419	0.658*	0.035	0.819**	—					
ToM15 understand	0.292	0.690*	0.665*	0.305	0.540	-0.153	0.697*	0.854**	—				
Affective ToM	0.201	0.252	0.638*	-0.159	0.123	-0.199	-0.055	0.354	0.442	—			
Aff.ToM understand	-0.350	-0.159	0.020	0.356	0.247	0.468	0.672*	0.571	0.219	-0.059	—		
Basic ERT	0.129	0.775**	0.370	0.546	0.559	-0.100	0.639*	0.669*	0.858**	0.201	0.139	—	
Complex ERT	-0.007	0.287	0.035	0.545	0.065	-0.222	0.378	0.297	0.327	0.080	0.240	0.659*	

Note. * *p* < .05, ** *p* < .01, *** *p* < .001

The psychosexual development is significantly correlated with the ability to recognize basic emotion (*r* = .77) and to understand the stories from the ToM15 task (*r* = .69).

Social development is significantly correlated with the performance of the Affective Theory Mind (*r* = .64) and with the ability to understand the stories from the ToM15 task (*r* = .66).

Regarding the dimensions of social cognition, the ability to recognize basic emotions is correlated with the performance of the first order ToM (*r* = .64), with the 2nd order ToM performance (*r* = .67) and with the ability to recognize complex emotions (*r* = .66). It is also correlated with the understanding of stories from the ToM15 task (*r* = .86).

The three indicators from the ToM15 task correlate with one another: understanding the stories, inferring the 1st order and the 2nd order mental states of others.

As a post-hoc observation, we note that Substance Abuse is significantly correlated with the 2nd order cognitive Theory of Mind performance (*r* = .66).

## Discussion

Previous research had shown that people with galactosemia have a lower well-being and psychosocial development than the control population and people with some other metabolic diseases [9, 10, 15]. Two previous research also showed some deficits in emotion recognition [24, 25] and theory of mind [25]. We wanted to replicate these findings and to deepen the description of possible deficits in these processes. We also investigated whether social cognition processes would be related to psychosocial development and mental well-being.

We hypothesised that our participants with galactosemia would show deficits in social cognition processes compared to control participants. We tested our hypothesis by comparing the scores of 11 patients with galactosemia and 31 control individuals using various neuropsychological assessment tools. We indeed found deficits in four validated social cognition measures: we showed that galactosemia is associated with deficits in cognitive theory of mind, and in affective theory of mind, in basic and complex emotion recognition. Finally, we found for these patients a positive link between emotion recognition and psychosexual development and a positive link between affective theory of mind and social development.

### Specific features of theory of mind in our sample with galactosemia

We used a well-validated tool to assess the cognitive theory of Mind [27]. Participants with galactosemia showed deficits in the 2nd order ToM but not in the 1st order ToM. These deficits showed even though we controlled participants correctly understood the stories. Our participants with galactosemia are thus able to build a 1st order theory of mind which is fundamental to basic social relations. A good first-order theory of mind helps adapting to an interlocutor notably when the situation is one-to-one. The 2nd -order theory of mind is more complex, notably because it requires more executive functions (i.e., working memory) and metacognition [39]. Deficits in the second-order theory of mind may hinder interactions when there is a third person involved. Being able to infer what a conversation partner knows/believes about a third party is important because one can predict and explain the behaviour of conversation partners based on what they know/feel. When the social situation exceeds the ability to deal with the different points of views, individuals may feel uneasy and overwhelmed leading to shyness and avoidance [12, 14]. Future research is needed to test whether the social avoidance observed in the galactosemia phenotype could be explained by deficits in second-order theory of mind.

We found that participants with galactosemia globally underperformed on the affective Theory of Mind task compared to controls. When digging deeper, we found they were able to differentiate situations with and without faux-pas, they correctly detected when a ‘faux-pas’ happens, they understood the beliefs of the protagonists (related to their good performance in 1st -order cognitive theory of mind), they were able to explain what was inappropriate and the intentions of the characters: they seem to be aware of social rules and conventions. Future research involving specific tests of social knowledge like the Test of Situations [40] would be interesting to be able to establish the affective theory of mind ability of people with galactosemia, controlling for their social knowledge. However, our participants had significantly more difficulties explaining what the feelings of the characters were. This indicates that they may not be able to show an appropriate reaction to the emotions of the social partner, not being able to empathise with them. This result replicates the deficit in affective empathy found by Hermans et al. [25]. Future replication with other mixed tests of theory of mind, like the combined stories test [40] would be interesting to precisely describe the types of mental states (i.e., epistemic, affective, or volitional mental states) that are difficult to infer for people with galactosemia. This knowledge is fundamental to tailor interventions improving social cognition skills in that population.

We found that, apart from the cognitive and affective ToM performance, participants with galactosemia had deficits in understanding the stories. We believe this is related to the global cognitive deficits found in galactosemia and the large distribution in our sample is congruent with the diversity usually found in cognitive tasks for people with galactosemia. We did not have the means to measure the IQ of our participants and we wonder whether the ability to understand the stories in the ToM15 and Faux-Pas tasks could be a proxy of intellectual level. Although it is reasonable to claim that the intellectual level would predict the understanding of the scenarios, more research is needed to assess how much of a proxy the understanding of scenarios can be to the intellectual level. Future research involving measures of both intellectual functioning and social cognition would help disentangle the respective impact of these domains on the deficits in social functioning. Hermans et al. [25] found little correlations between social cognition and intellectual functioning except with global intelligence. A large sample is required to have enough statistical power to perform multiple regressions.

### Specific features of emotion recognition deficits in our sample with galactosemia

We used two different tools to assess emotion recognition. The first measure was a dynamic and timed task with basic facial emotion expressions. Participants with galactosemia showed deficits in that task, with a major impairment in fear, anger and disgust. Korner et al. [24] found specific deficits in anger, disgust, fear and surprise. Hermans et al. [25] found specific deficits in disgust, fear, happiness, and sadness. The recognition of disgust and fear was consistently found to be impaired in the three studies. Recognition of anger was found to be impaired in two studies. All three studies used different tasks and stimuli, with their own strengths and weaknesses, which may explain the differences between emotions. We think it is clear that galactosemia is associated with emotion recognition deficits but the type of emotions impaired is yet to confirm. A large multinational study and a meta-analysis could provide solid knowledge.

However, given the deficit in visual information processing associated with galactosemia [24], one could argue that participants with galactosemia have had issues mostly because the stimuli is very short. It might not be emotion recognition per se that is impaired but more generally rapid visual information processing (including emotions). However, the complex emotion recognition task we used, which was not timed, gives us a hint that emotion recognition is impaired per se. Participants with galactosemia underperformed and their performance was not increased when they spent longer time on the task. It is thus not only a problem of processing speed. We acknowledge that the Reading the Mind in the Eyes Test has another complexity related to language that may have inflated the deficits found in our participants though people with galactosemia do not usually have issues with receptive language and vocabulary. However, we note that some participants found it difficult and future research using the Child RMET (the same task with adapted vocabulary) would be more appropriate to confirm our findings.

Consistent with models of social cognition [19], we found few correlations between the different social cognition measures, among the galactosemia group, indicating that they are distinct dimensions and processes.

Importantly, the ToM and emotion recognition deficits are hurdles to smooth social interactions that require remediations. There are a growing number of evidence-based remediations for social cognition, targeting theory of mind and/or emotion processing (i.e., ToMRemed, RC2S) and could thus be indicated for patients with galactosemia. We contend that early referral to social cognition and mental health specialists should be included in the care and follow-up guidelines for galactosemia.

### Well-being and psychosocial development

Previous research showed that patients with galactosemia were delayed in their psychosocial development and had lower mental health than the general population. We tried to replicate these findings using the same psychosocial development measure as Maurice-Stam et al. [15] and a validated mental health tool (the WEMWBS). We also intended to test whether psychosocial and mental health are related to social cognition processes.

Consistent with previous research, we found that participants with galactosemia were significantly delayed compared to control participants in psychosexual development. For participants with galactosemia, their psychosexual development was highly correlated with their (impaired) basic emotion recognition performance and (impaired) understanding of the ToM15 stories. It appears that this social cognition process (emotion recognition) is related to psychosexual development in our sample and future research is needed to replicate and strengthen this finding. Also, another measure of intimate relationship quality (marital status, number of partners, length of relationships, etc.) might be more informative than simply the age of intimacy onset.

We found marginally lower mental health in participants with galactosemia compared to control participants. The average mental health score for participants with galactosemia was 4 points lower than the control score. This result is consistent with the recent paper by Welsink-Karssies et al. [41] where they measured anxiety and depression. People with galactosemia appear to have a lower mental health but the difference with the control population is not significant. Both our study and the one by Welsink-Karssies and colleagues [41] suffer from a small sample and hence a poor statistical power to detect such a small effect. On another note, we did not find correlations between mental well-being and social cognition measures. If there were a relation between mental health and social cognition processes, it might be indirect via intermediate processes we did not address in this study like positive and negative affectivity, social self-esteem and self-efficacy, the feeling of loneliness, the quality of social relations [42–46]. Future research is warranted to investigate a possible contribution of social cognition processes (and deficits) to mental health in galactosemia.

Finally, contrary to what has been found in the literature, our participants with galactosemia were not delayed in their social development. It appears that they reached the milestones just like control participants. Our sample is not representative of the whole galactosemia population. In fact, we recruited them through online messages, so they would have to be quite independent and socially integrated to be aware of our study and decide to participate. However, social development in our galactosemia sample was positively correlated with the (deficient) affective Theory of Mind performance. We think that affective ToM deficit is thus a risk factor to a lower social well-being in galactosemia and additional research is needed. Furthermore, the course of Life questionnaire does not fully address the question of social functioning and well-being. It gives a good picture of the social trajectory with galactosemia. We contend that other tools specifically designed to measure social functioning and well-being would be relevant in future studies. (e.g., social self-esteem and self-efficacy, feeling of loneliness, quality of social relations).

### Strengths and Limitations

Our study used social cognition tools that complement previous social cognition studies in a galactosemia sample. We were able to provide new insights into theory of mind and emotion recognition impairments. Obviously, these results need replication, notably with a larger sample, and more knowledge about the characteristics of the participants. Our results should not be generalised and this study does not give a definitive answer to social cognition skills in this condition. However, it participates in cumulative research. Most researchers in the field of rare diseases are faced with the difficulty of accessing patients, and though we all would prefer to lead large-scale studies, small studies help to prune the various avenues to be investigated before investing time, effort and money in large-scale costly research.

## Conclusion

The puzzle of long-term complications in galactosemia must be thoroughly described and explained so that patients can receive relevant and evidence-based interventions. In this paper, we give some evidence that social cognition is diffusely impaired in a sample of French adults with galactosemia. We contend that early interventions are indicated to increase psychosocial skills and prevent social and well-being decrements to allow patients with galactosemia to achieve a better quality of social life [47].

## Electronic supplementary material

Below is the link to the electronic supplementary material.

Supplementary Material 1

## Data Availability

Supplementary material and data is available on https://osf.io/3mw2x/. All material and data can be sent on request to the first author.

## Footnotes

1 It did not appear relevant in this study for our sample to add any unconventional gender identities. We reasoned that fluid and non-binary individuals would be very rare in our sample and that it would add an unnecessary complexity.

2 The unilateral hypothesis tests whether control participants perform better than participants with galactosemia.
