# Supplementary material for: Social cognition, psychosocial development and well-being in galactosemia
Source: Orphanet J Rare Dis. 2024 Sep 6;19:325. doi: 10.1186/s13023-024-03335-2 (PMC11378408; doi:10.1186/s13023-024-03335-2)
Supplement: Supplementary file 1 — Supplementary Material 1 [file 13023_2024_3335_MOESM1_ESM.docx]

# Supplementary Material A – tests and questionnaires

## Warwick-Edinburgh Mental well-being Scale

Trousselard, M., Steiler, D., Dutheil, F., Claverie, D., Canini, F., Fenouillet, F., ... & Franck, N. (2016). Validation of the Warwick-Edinburgh mental well-being scale (WEMWBS) in French psychiatric and general populations. *Psychiatry research*, *245*, 282-290.

### Example of item

*Durant les deux dernières semaines, je me suis senti(e) optimiste quant à l’avenir*

*Jamais / rarement / parfois / souvent / tout le temps*

## Theory of Mind – 15

Desgranges, B., Laisney, M., Bon, L., Duval, C., Mondou, A., Bejanin, A., ... & Muckle, G. (2012). TOM-15: Une épreuve de fausses croyances pour évaluer la théorie de l’esprit cognitive. *Revue de neuropsychologie*, (3), 216-220

### Example of first-order vignette, ToM question


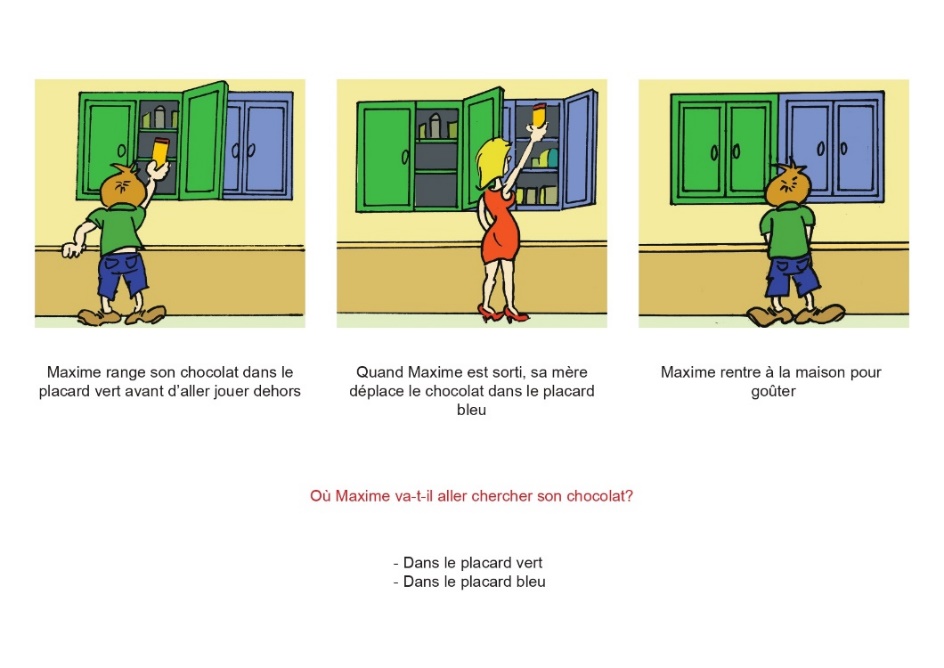


### Example of first-order vignette, comprehension question


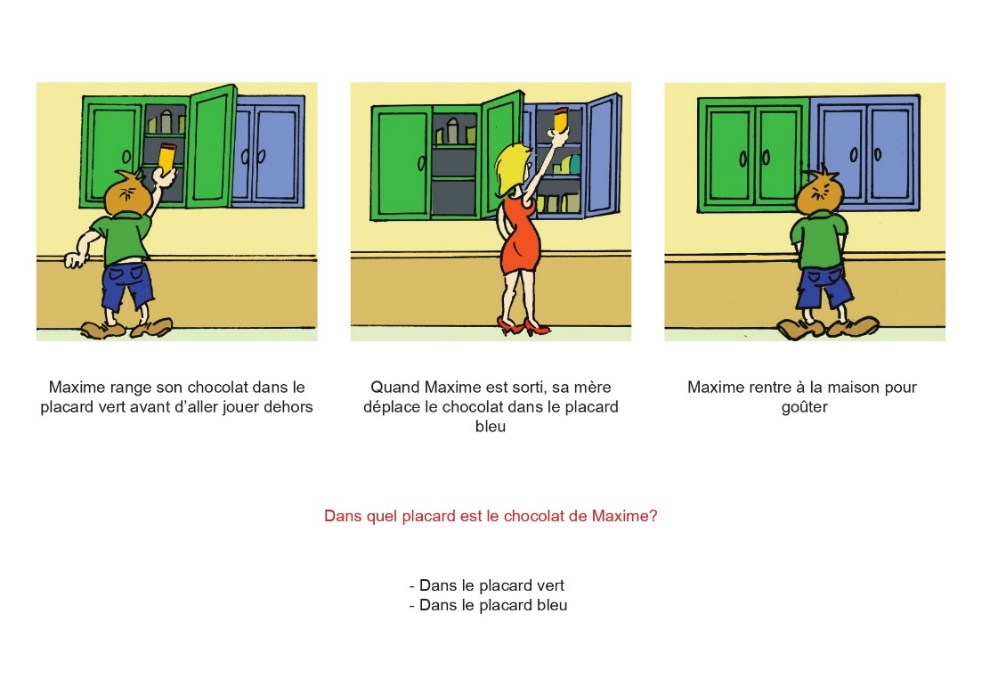


### Example of second-order vignette, ToM question


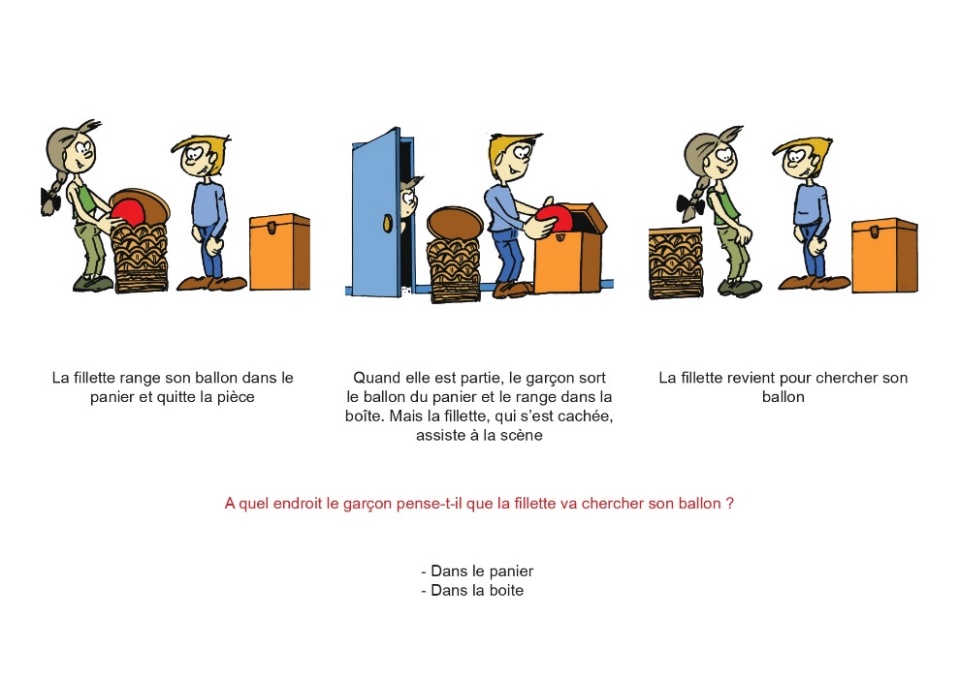


### Example of second-order vignette, comprehension question


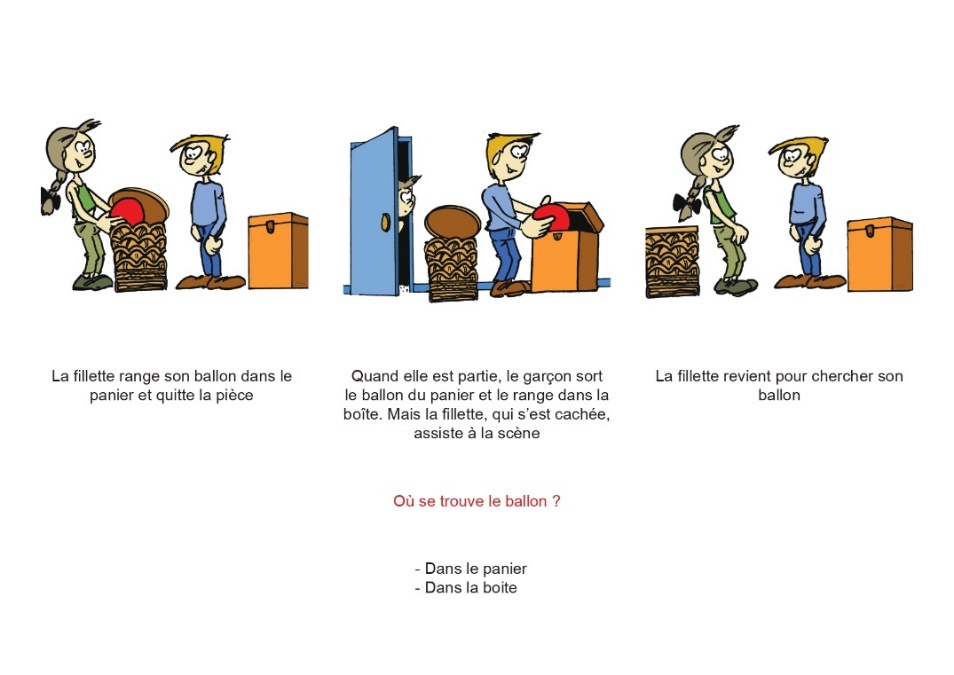


## Course of Life questionnaire – examples of items

Grootenhuis, M. A., Stam, H., Destrée-Vonk, A., Heymans, H. S. A., & Last, B. F. (2003). Levensloop Vragenlijst voor Jong-Volwassenen. *Gedrag & Gezondheid: Tijdschrift voor Psychologie en Gezondheid*

| Life period | dimension | Item | Response options | coding |
| --- | --- | --- | --- | --- |
| Childhood | Social | Enfant, avez-vous participé pendant au moins un an à une activité sportive en club telle que le football, le judo ou l'équitation ? | Oui / non | 1 / 0 |
| Childhood | Social | Enfant, avez-vous participé pendant au moins un an à une activité extra-scolaire telle que les scouts, le théâtre, la musique ? | Oui / non | 1 / 0 |
| Childhood | Autonomy | Enfant, aviez-vous un travail quelconque en dehors de votre domicile pour gagner de l'argent, par exemple en lavant des voitures, en tondant des pelouses, en faisant du baby sitting ? | Oui / non | 1 / 0 |
| Teenage | Social | Adolescent(e), avez-vous participé à des activités périscolaires (organisées au sein de l'école : club de théâtre, association sportive,  club de poterie…)? | Oui / non | 1 / 0 |
| Teenage | Social | Adolescent.e, combien d'amis (fille ou garçon) aviez-vous habituellement ? | Aucun / 1/ 2 ou 3 / 4 ou plus | 0 / 1/ 2/ 3 |
| Teenage | Substance abuse | Adolescent.e, avez-vous consommé de l'alcool ? | Jamais / occasionnellement / souvent / très souvent | 0 / 1/ 2/ 3 |
| Adulthood | Substance abuse | Adulte, avez-vous consommé des drogues douces (shit, beuh, joint,  marijuana, haschisch...) ? | Jamais / occasionnellement / souvent / très souvent | 0 / 1/ 2/ 3 |
| Adulthood | Substance abuse | Adulte, avez-vous joué pour de l'argent (par exemple, paris en ligne,  poker en ligne, dans un casino ou à une machine à sous) ? | Jamais / occasionnellement / souvent / très souvent | 0 / 1/ 2/ 3 |
| Whole life | Psychosexual | A quel âge avez-vous eu votre 1er petit copain / 1ère petite copine? | Je n’ai encore jamais eu de petit.e-ami.e / entre 12 et 14 ans / entre 15 et 17 ans/ après 18 ans | 0 / 3/ 2/ 1 |
| Whole life | Autonomy | A quel âge avez-vous quitté le domicile de vos parents pour vivre  ailleurs ? | J’habite toujours chez mes parents / avant 11 ans / entre 11 et 15 ans / entre 16 et 20 ans / après 21 ans | 0 / 4/ 3 /2 / 1 |
| Whole life | autonomy | A quel âge êtes-vous parti.e en vacances sans parents, sans  accompagnateurs et sans adultes responsables pour la 1ère fois ? | Je ne suis encore jamais parti en vacances sans responsables / entre 12 et 14 ans / entre 15 et 17 ans / entre 18 et 20 ans / après 21 ans | 0 / 4 /3 /2 /1 |

## Reading the Mind in the Eyes Test

Prevost, M., Carrier, M. E., Chowne, G., Zelkowitz, P., Joseph, L., & Gold, I. (2014). The Reading the Mind in the Eyes test: validation of a French version and exploration of cultural variations in a multi-ethnic city. *Cognitive neuropsychiatry*, *19*(3), 189-204.

### Example of item

*Jaloux / paniqué / arrogant / haineux*

## Faux-Pas Test

Baron-Cohen, S., O’Riordan, M., Jones, R., Stone, V., & Plaisted, K. (1999). A new test of social sensitivity: Detection of faux pas in normal children and children with Asperger syndrome. *Journal of Autism and Developmental Disorders*, *29*(5), 407-418.

### Control scenario and questions

Julie se trouve à une fête chez son ami Olivier. Elle parle à Olivier quand une autre femme s'approche d'eux. C'est une des voisines d'Olivier. La femme dit: "bonjour!" puis elle se retourne vers Julie et ajoute: "je ne pense pas que nous nous connaissons, je m'appelle Marie, et vous?"; "Julie!" répond-elle. A ce moment, Olivier demande: "quelqu'un veut-il quelque chose à boire?"

| item | responses | coding |
| --- | --- | --- |
| Est-ce que quelqu'un a dit quelque chose qu'il n'aurait pas dû dire ou a dit quelque chose de maladroit? | Oui /non | 0 / 2 |
| *The next questions are not shown unless the participant answers yes to the first question (which is mistake)* | | |
| Qui a dit quelque chose qu'il n'aurait pas dû dire ou a dit quelque chose de maladroit ? | Julie / Olivier | 0 |
| Pourquoi il/elle n'aurait pas dû le dire ? Ou pourquoi était-ce maladroit? | Free answer | 0 |
| Pourquoi pensez-vous qu'il/elle l'a dit ? | Free answer | 0 |
| Olivier savait-il que Julie et Marie ne se connaissaient pas ? | Free answer | 0 |
| Qu'est-ce que Julie a ressenti selon vous? | Free answer | 0 |
| *The next questions are comprehension questions that are systematically asked even when they give wrong answers before* | | |
| Dans l'histoire, où était Julie ? | Free answer | 1 (chez Olivier) / 0 |
| Est-ce que Julie et Marie se connaissaient déjà ? | Oui / non | 0 / 1 |

### Faux-Pas scenario and questions

Le mari d'Hélène organise une fête surprise pour l'anniversaire de sa femme. Il a invité Sarah, une amie d'Hélène, en lui disant: "ne le dis à personne, surtout pas à Hélène." La veille de la fête, Hélène se trouvait chez Sarah quand celle-ci renverse du café sur sa nouvelle robe qui était accrochée à sa chaise. "Oh!" dit Sarah, "j'allais la porter à ta fête!". Hélène répondit alors: "Quelle fête?" "Bon" dit Sarah "Allons voir si nous pouvons enlever cette tâche."

| Est-ce que quelqu'un a dit quelque chose qu'il n'aurait pas dû dire ou a dit quelque chose de maladroit? | Oui /non | 1 / 0 |
| --- | --- | --- |
| *The next questions are not shown unless the participant answers yes to the first question (which is the correct answer)* | | |
| Qui a dit quelque chose qu'il n'aurait pas dû dire ou a dit quelque chose de maladroit ? | Hélène / Sarah / le mari d’Hélène | 0 / 1 / 0 |
| Pourquoi il/elle n'aurait pas dû le dire ? Ou pourquoi était-ce maladroit? | Free answer | 1 / 0 |
| Pourquoi pensez-vous qu'il/elle l'a dit ? | Free answer | 1 / 0 |
| Sarah s'est-elle rappelée que la fête était une surprise ? | Free answer | 1 / 0 |
| Qu'est-ce que Hélène a ressenti selon vous? | Free answer | 1 / 0 |
| *The next questions are comprehension questions that are systematically asked even when they give wrong answers before* | | |
| Dans l'histoire, pour qui est organisée la fête surprise ? | Free answer | 1 ( Hélène) / 0 |
| Qu'est-ce qui a été renversé sur la robe ? | Free answer | 1 (du café) / 0 |

## Emotion recognition Task (KDEF-Dyn)

Calvo, M. G., Fernández-Martín, A., Recio, G., & Lundqvist, D. (2018). Human observers and automated assessment of dynamic emotional facial expressions: KDEF-dyn database validation. *Frontiers in psychology*, *9*, 2052.

### Example of a happy stimulus

<https://drive.google.com/file/d/1yhYo_MiD5yO73r_Cg5BVoX0u5Uw3a5U_/view?usp=drive_link>
